# Development of a lifestyle intervention using the MRC framework for diabetes prevention in people with impaired glucose regulation

**DOI:** 10.1093/pubmed/fdv110

**Published:** 2016-10-17

**Authors:** Jacqui Troughton, Sudesna Chatterjee, Siân E. Hill, Heather Daly, Lorraine Martin Stacey, Margaret A. Stone, Naina Patel, Kamlesh Khunti, Thomas Yates, Laura J. Gray, Melanie J. Davies

**Affiliations:** 1 Leicester Diabetes Centre, University Hospitals of Leicester, Leicester General Hospital, Gwendolen Road, Leicester LE5 4PW, UK; 2 Diabetes Research Centre, University of Leicester, Leicester General Hospital, Gwendolen Road, Leicester LE5 4PW, UK

**Keywords:** complex intervention, diabetes prevention, impaired glucose regulation, structured education

## Abstract

**Background:**

We report development of a group-based lifestyle intervention, Let's Prevent, using the UK Medical Research Council (MRC) framework, and delivered by structured education to prevent type 2 diabetes mellitus (T2DM) in people with impaired glucose regulation (IGR) in a UK multi-ethnic population.

**Methods:**

Diabetes Education and Self-Management for Ongoing and Newly Diagnosed (DESMOND) is the first national T2DM programme that meets National Institute for Health and Care Excellence criteria and formed the basis for Let's Prevent. An iterative cycle of initial development, piloting, collecting and collating qualitative and quantitative data, and reflection and modification, was used to inform and refine lifestyle intervention until it was fit for evaluation in a definitive randomized controlled trial (RCT). The programme encouraged IGR self-management using simple, non-technical language and visual aids.

**Results:**

Qualitative and quantitative data suggested that intervention resulted in beneficial short-term behaviour change such as healthier eating patterns, improved health beliefs and greater participant motivation and empowerment. We also demonstrated that recruitment strategy and data collection methods were feasible for RCT implementation.

**Conclusions:**

Let's Prevent was developed following successful application of MRC framework criteria and the subsequent RCT will determine whether it is feasible, reliable and transferable from research into a real-world NHS primary healthcare setting.

**Trial Registration:**

ISRCTN80605705.

## Introduction

Screening studies carried out in primary care in the UK^1^ have shown that screening for type 2 diabetes mellitus (T2DM) will identify around 15% of middle aged adults with impaired glucose tolerance or impaired fasting glucose, collectively termed impaired glucose regulation (IGR) and also known as pre-diabetes mellitus.^2^ IGR is considered to be a high-risk state for T2DM, cardiovascular disease (CVD) and increased mortality^3,4^ and is now referred to as ‘at high risk of developing diabetes’ under new guidelines on terminology.^5^

There is evidence that, in people with IGR, lifestyle modifications can substantially reduce the risk of developing T2DM.^6^ Screening and lifestyle interventions are also likely to be cost-effective.^7^ The Diabetes Prevention Programme^8^ and the Finnish Prevention study^9^ showed that lifestyle programmes addressing weight loss, healthy diet and physical activity reduce the risk of developing T2DM in those with IGR by 58%. Successful lifestyle programmes have also been carried out in many other countries, including India,^10^ China^11^ and Japan.^12^

There is limited evidence, however, regarding the feasibility of translating this research into practice. Many research-based prevention programmes have involved multiple one-to-one counselling sessions. The Diabetes Prevention Programme had a median of 20 individual counselling sessions over a 4-year period.^9^ The intensity of such an intervention, even if cost-effective in the long term, is likely to place acute strain on healthcare resources in the short term. Even resource-rich countries, such as Germany and Finland, where diabetes prevention is a public health priority, have been unable to replicate the intensive nature of diabetes prevention programmes when implemented into clinical practice.^13,14^

Ethnic minorities present a further challenge. The high risk of diabetes and premature heart disease in South Asians (SAs),^15^ the diversity of dietary practice and reported lower levels of moderate physical activity^16^ in subgroups of SA, make it crucial to develop a flexible and culturally informed intervention. At the time of devising this programme there were no reported studies on the prevention of diabetes in migrant populations in the UK. Subsequently, the PODOSA study, which recruited SA participants to a lifestyle intervention trial, confirmed the success of a personal and community-orientated approach to recruitment in this ethnic group.^17^ New guidelines from the National Institute for Health and Care Excellence (NICE) state that those identified with a moderate or high risk of developing T2DM should be offered culturally appropriate information or support, in a range of formats and languages, including structured education, to help them change their lifestyle.^5^

Structured education refers to group-based patient-centred educational programmes that have a clear philosophy and a written curriculum with supporting resources, are underpinned by appropriate learning and psychological theory, and are evidence-based and delivered by trained, quality assessed educators.^18^ Such programmes have been recommended by NICE as a cost-effective method of improving glycaemic control and quality of life in people with T2DM^5^ and have been shown to be feasible to implement within the NHS at national level.^19^

Diabetes Education and Self-Management for Ongoing and Newly Diagnosed (DESMOND) is the first national programme for T2DM that meets NICE criteria^20,21^ and a randomized controlled trial (RCT) has demonstrated its effectiveness in improving weight, smoking behaviours and illness beliefs.^21^ The DESMOND model is based on an empowerment philosophy that sees the participant as capable and responsible for their own health decisions and behaviours.^22^ This philosophy uses non-directive educational methods and recognizes that individuals with IGR have insight and expertize in relation to their own food choices.

**Fig. 1:**
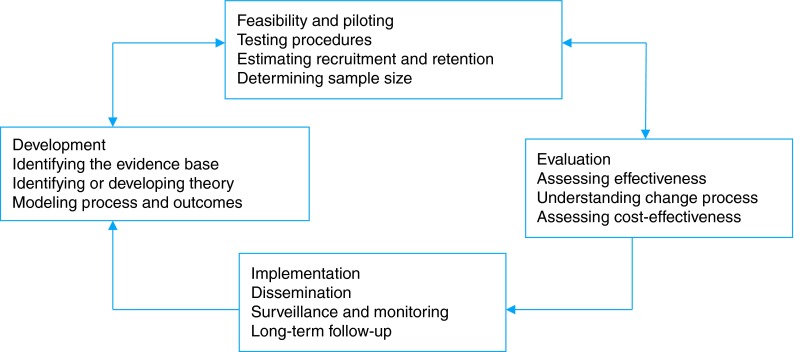
MRC framework for the evaluation of complex interventions.^23^

The UK Medical Research Council (MRC) Framework for Complex Interventions to Improve Health has internationally recognized criteria to guide the development and evaluation of health behaviour change programmes. The most recent MRC guidance suggests including the following phases: development, feasibility and piloting, evaluation and implementation^23^ (Fig. 1).

This framework suggests that an early task is the development of a theoretical understanding of the likely process of change with the planned intervention by drawing on existing evidence and theory. Bartholomew's intervention mapping^24^ identified the following theories as appropriate for underpinning the intervention: the Common Sense Model of Illness,^25^ Social Learning Theory,^26^ Gollwitzer's Implementation Intentions Theory^27^ and Dual Processing Theory.^28^ The DESMOND model is also underpinned by these adult learning theories and psychological models of learning.

There is an urgent need to design and test prevention programmes that meet the needs of ethnically diverse communities and are feasible, cost-effective and replicable in a real-world UK healthcare setting. The lifestyle intervention described was developed to meet this need and is currently being formally evaluated in an RCT.^29^ We demonstrate in this article how the MRC framework criteria were used to develop a group-based lifestyle intervention known as Let's Prevent to prevent T2DM in people identified as being ‘at risk’. Specifically, we have focused on the development and feasibility/piloting phases of the process.

## Methods

### Study design and recruitment

The Let's Prevent intervention was developed by a core multi-disciplinary team in collaboration with the DESMOND collaborative and the National Physical Activity Centre in Loughborough. It was designed as a group educational programme with a written curriculum suitable for the broadest range of participants, to be deliverable in a community setting for ease of access for patients, and to have the potential for integration into future routine clinical care. The session content was founded on a sound evidence base and guided by a review of the literature surrounding nutrition, exercise and educational principles with pragmatic decisions on best practice where the evidence was ambiguous, lacking or conflicting.

Using the DESMOND model, the programme was ∼6 h long, deliverable in either one full day or as two half-day equivalents. It was designed to be facilitated by two trained healthcare professionals (educators) to a group of between 5 and 12 participants with IGR, who had the option of bringing an accompanying person. It was delivered as two versions. The first was broadly suitable for most White European groups, referred to as the Let's Prevent programme, and the second was adapted to suit SA patient groups and referred to as the SA Let's Prevent programme.

Both versions of the educational intervention and the educator training programme were modelled and refined using an iterative process, including piloting, feedback, analysis, reflection and modification, based on a methodology previously developed for modifying an educational programme.^30^ Key to the process was the collection and analysis of qualitative and quantitative data. A degree of objectivity and a high level of rigour were attained by involving academic researchers in this process.

The intervention was informed by qualitative findings^31^ that individuals with IGR expressed limited understanding of their diagnosis, its physical consequences and subsequent management. Individuals often demonstrated unsound beliefs about their diagnosis because of flawed appraisals made in the absence of concrete information. Many had no prior understanding of IGR or their risk of CVD, felt confused by the diagnosis and wanted clarification of what was meant by their status as ‘high risk’. Respondents consistently expressed the need for support and education at diagnosis. Written support alone was not valued but healthcare professional time was seen as beneficial. A group educational approach was considered acceptable to the sample. These findings are consistent with another qualitative study in the UK where individuals with IGR were found to want a clear explanation of the illness processes and were in line with the Leventhal Common Sense Model.^32^

Qualitative data were gathered to ascertain the experiences of both participants and educators of engaging in the programme. Data were collected via observation, telephone and face-to-face interviews and focus groups. Flexible topic guides were developed by trainers and qualitative researchers to inform and facilitate the qualitative data collection process.

The SA Let's Prevent programme was developed concurrently to address the needs of the SA population living in the UK, many of whom do not speak English. Core educational messages were identical to Let's Prevent but changes were made to ensure that food and activity messages were culturally relevant. Teaching resources (images and models) were developed to ensure that the programme was not reliant on the written word. A previous action research project^30^ had identified a need for substantially increased delivery time when interpreters were used (four 3-h sessions). Table 1 gives an overview of the content and theoretical underpinning of the programme, as well as the duration for specific sections.

**Table 1:** Content of the Let's Prevent programme

	*Theory*	*Sample activity*	*Duration (min)*
Session 1			
Introduction	—	—	10
Patient story	CSM, DPT	Participants are asked to tell their story about how they discovered they had IGR and their current knowledge of IGR (in relation to identity, cause, consequence, control/treatment and timeline Uses participants' stories to support them in learning how the body regulates glucose and signs symptoms, causes, controllability and timeline of IGR	50
Taking control 1 Weight management	CSM, DPT, SLT	Uses participants' stories to support them in discovering how weight/waist affects IGR Provides knowledge and skills for food choices to control weight and self-regulation	30
Physical activity	CSM, DPT, SLT	Uses participants' stories to support them in discovering how physical activity affects IGR and overall health. Provides knowledge and skills for activity choices to manage IGR Pedometers given out to set personalized goals and support self-regulation	40
How am I doing?	SLT	Participants reflect on what issues have come up in the programmes so far	5
Session 2			
Reflections	SLT	Participants reflect issues that have arisen in the programme so far	10
Professional story	CSM	Uses participants' stories to support them in discovering that IGR is a risk factor for large blood vessel damage, e.g. CVD and stroke. Uses stories to discover IGR is associated with other risk factors (e.g. blood pressure and cholesterol). Facilitates participants to work out how they can reduce these risk factors and prevent complications	30
Taking control 2 Food choices: focus on fats	DPT, SLT	Provides knowledge and skills for food choices to reduce cardiovascular risk factors. Uses food models and labels to provide mastery experiences. Provides opportunity for vicarious learning	50
Self-management plan	SLT	Participants supported in developing personal self-management plans	30
Questions	CSM	Checks that all questions raised by participants throughout the programme have been answered and understood	40
What happens next?	SLT	Follow-up care outlines	5

CSM, common sense model; DPT, dual processing theory; SLT, social learning theory.

The nutritional goals from the Diabetes Prevention Programme^8^ and the Finnish Prevention Study^9^ and the physical activity goals from the PREPARE study^33,34^ were incorporated into the Let's Prevent programme. These goals were to aim for a sustained weight reduction of >5% body weight, a moderate reduction in total fat of <30% energy intake, a low saturated fat intake of <10% energy intake, higher fibre intakes of >15 g per 1000 calories and a minimum recommendation of 30 min of moderate-intensity physical activity per day.

The pilot sessions were categorized as developmental work rather than formal research and using participants from the ADDITION diabetes screening study.^35^ This work was not, therefore, subject to ethics committee approval or formal consent procedures, although all those who participated were aware of the context of the sessions that they attended and had agreed verbally to contribute to the development process. For this reason, we report quantitative findings in broad terms and qualitative findings without using direct quotations.

Between October and November 2007, 83 individuals with English as their first language were identified as having been diagnosed with pre-diabetes in the past 12 months. An invitation letter was sent to all 83 individuals inviting them to take part in the pilot study, which was followed up by a telephone call a week later. Thirty-eight individuals (46% of those identified) responded favourably to the initial invitation, while 35 actually attended the pilot programme (42%). The most common reasons for not wanting to attend were being unable to take time off work and ill-health.

Participants were provided with a handbook containing a summary of the content of the programme and resources for plotting their risk profile based on their biochemical and anthropometric data. Tape measures and pedometers (SW 200, Yamax Corporation, Tokyo, Japan) were distributed to support self-regulation. Providing a pedometer as part of an education programme has been found to be effective in improving glucose tolerance in those with IGR.^33,34^

Quantitative data collection pre- and post-intervention included pedometer step counts and the validated questionnaires Dietary Instrument for Nutrition Education (DINE),^36^ Brief Illness Perception Questionnaire^37^ and International Physical Activity Questionnaire (IPAQ).^38^ Pedometer use encouraged goal-setting and facilitated monitoring of physical activity levels. The physical activity target was to increase daily step count by 4500 steps, which is equivalent to ∼45 min of walking. It was suggested that this goal should be broken down into smaller more achievable goals such as increasing steps by 500 a day every fortnight, in order to provide sufficient time for participants to adjust to their new level of activity.

Qualitative data were collected by researchers through observation, interviews and focus groups. Views were considered from a range of stakeholder groups, including people with IGR, trainers, healthcare professional educators and interpreters. The main topics discussed are summarized in Table 1.

Following the analysis of all data, there was a period of reflection to consider the implications of the findings. Based on these findings, refinements were made to the curricula, resources and training packages.

A report by NICE in 2003^20^ suggests many self-management programmes for long-term conditions have variable outcomes. One reason is the variable nature of the delivery of the programmes by healthcare professionals. To ensure treatment fidelity, a training programme with supporting manual and resources and a quality development (QD) programme were developed. The QD programme consisted of both internal and external processes. The internal process encouraged the educators to reflect on their practice using self-reflection sheets and peer reflection sheets as tools for personal development.

The external components of the QD programme involved an observation sheet and observer tool (involving the assessor having to record a mark every 10 s of who was talking in the room). These external QD components provided quantitative data on content and process indicators, to support not only educator practice but to be used if required for the interpretation of study outcomes.^39^

### Statistical analysis

It was recognized that our sample was not powered to give meaningful outcomes but would give us some guidance on the direction of the results. Data analysis was undertaken using SPSS v16.0 software. Paired sample *t*-tests were used to test for within group differences between the pre- and post-programme data measurements if the data were normally distributed and homogeneity of variance was assumed. Non-parametric data were analysed using Wilcoxon's signed ranks test.

## Results

The findings are reported to demonstrate the way in which they contributed to the development process by guiding our reflections about further modifications needed to the intervention and determination of the point at which the intervention was considered to be fit-for-purpose for the RCT.

In total, six healthcare professionals and four interpreters were trained to deliver the interventions. Two development cycles were carried out for both the Let's Prevent programme and SA Let's Prevent programme to identify and implement modifications until all components of the intervention were considered fit for purpose and ready for formal evaluation.

Piloting for these two cycles involved delivery of the programme to a total of 49 participants from primary care identified as having IGR, living in Melton Mowbray in Leicestershire. The mean age of participants was 65.83 ± 6.6 years and 34% were female. On average, participants were significantly older in the group that responded positively (mean 65.34 years, SE 1.06) compared with those that did not respond or responded negatively (mean 60.11 years, SE 1.33, *P* < 0.05). There was a significant association between participant's gender and whether or not they responded positively (*P* < 0.05). The odds ratio indicated that participants were more likely to attend if they were male.

Three pilot Let's Prevent programmes were delivered, two in the half-day format both with 11 participants and one in the full day version with 13 participants. The interventions were delivered by two trained educators.

The SA Let's Prevent programme was piloted in two cycles in Leicester city to a total of 24 SA participants identified as having IGR, for whom English was not a first language. The mean age of these participants was 53.7 years and 57% were female.

### Phase 1 of the piloting cycle

Following the first development and piloting cycle of Let's Prevent, there was an increase in self-reported fibre intake and a decrease in self-reported total fat intake captured by the DINE questionnaire. Analysis of the illness perception questionnaires indicated that participants reported an increase in perceived knowledge of IGR and an increase in the perceived effectiveness of lifestyle change for controlling/treating their IGR. Although there was an increase in self-reported walking activity as measured by IPAQ questionnaires, no difference was seen between baseline and follow-up pedometer counts. Qualitative interviews identified that participants felt motivated to attend Let's Prevent to find out information about their condition and learn what they could do to reduce their risk of developing diabetes. Table 2 summarizes the baseline and follow-up data from Phase 1 of the pilot study.

**Table 2:** Phase one baseline and follow-up data

	*Number of complete data sets*	*Baseline value*	*Follow-up median*	*Significance (two-tailed)*
Perceived effect of IGT (consequences)^a^	23	1	3	0.01
Timeline associated with IGT^a^	22	4	3	0.30
Perceived control over IGT^a^	23	5	7	0.08
Perceived response efficacy of lifestyle change at treating IGT^a^	35	9	10	0.04
Perceived symptom load^a^	25	0	0	0.19
Concern at having IGT^a^	23	9	10	0.90
IGT coherence (knowledge)^a^	25	5	7	<0.01
Emotional representations^a^	25	1	1	0.45
Total fibre score	25	28	37	<0.01
Total fat score	24	19	18	0.21
Total unsaturated fat	23	9	10	0.13
Total walking activity (MET.minutes/week)	24	891	1386	0.01
Overall moderate-to-vigorous physical activity (MET.minutes/week)	23	2376	2772	0.99
Pedometer counts (steps per day)	14	5500	4700	0.97

^a^Questionnaire items used a 10-point Likert scale.

Overall, after attending the programme, participants reported a sense of empowerment and motivation to do something about their condition. When asked about key messages that they remembered, participants described these as losing weight and eating properly, more specifically reducing saturated fat and looking at types of food eaten. A lesser number spoke of the need to exercise more. Overall, qualitative and quantitative findings suggested a good level of fidelity for the intervention. As a result of both quantitative and qualitative feedback regarding physical activity, the curriculum was subsequently modified to address the suggestion that the physical activity section of the curriculum needed strengthening. This section was simplified, new educational resources were developed and educators received further training around physical activity messages.

Following the first development and piloting cycle of SA Let's Prevent, qualitative findings suggested that participants felt more confident about their knowledge and understanding of IGR and that they felt more likely to do something about it. When asked about the resources, participants responded favourably but also suggested that they would like more positive food messages that were culturally appropriate to them. The quantitative data collected from people attending the SA sessions were limited by the fact that the questionnaires were not translated into SA languages. The data that were collected suggested, however, that the intervention had led to a reduction in feelings of depression and anxiety and a trend towards increased walking activity (IPAQ questionnaire). Initial qualitative and quantitative findings were encouraging but, as a result of the qualitative findings, more food models of SA fruits and vegetables to promote positive food messages (such as okra, mango, aubergines, onions, spinach) were included.

Key changes implemented to education sessions for the SA population in the second cycle of the pilot included a revised and simplified physical activity section, inclusion of more Asian food models of fruit and vegetables, facilitation notes and prompts were added to promote links between certain sections of the curriculum, reinforce key messages, promote continuity and increase clarity. Presentation of risk factors and complications was revised and some changes were made to pre-course materials given to participants. Finally, there was some amendment of the action planning session for the SA curriculum and some organizational issues such as storage space for resources for education sessions were addressed.

### Phase 2 of the piloting cycle

The final programme was piloted after revisions as shown in Table 2. By the end of the second development and piloting cycle, it was clear from the qualitative and quantitative data collected that both programmes were proving to be highly successful at promoting positive food choices and increasing physical activity to levels that were consistent with minimum recommendation for health as well as targeting the psychological determinants of behaviour change. Self-reported walking activity, total energy expenditure and fibre intake all improved post-intervention. However, these differences were non-significant. Pedometer count and self-reported total fat intake were found to improve significantly post-intervention, and although the small sample size meant that the results cannot be generalized, it was nevertheless a pleasing indication of the success of the programme. Table 3 illustrates the baseline and follow-up data obtained from Phase 2 of the pilot study.

**Table 3:** Phase two baseline and follow-up data

	*Number of complete data sets*	*Baseline value*	*Follow-up median*	*Significance (two-tailed)*
Perceived effect of IGT (consequences)^a^	5	1	2	0.29
Timeline associated with IGT^a^	4	2	4.5	0.29
Perceived control over IGT^a^	5	5	7	0.16
Perceived response efficacy of lifestyle change at treating IGT^a^	5	9	10	0.59
Perceived symptom load^a^	5	0.5	2	0.14
Concern at having IGT^a^	5	6	8	0.68
IGT coherence (knowledge)^a^	5	1	6	0.10
Emotional representations^a^	5	1.5	5	0.20
Total fibre score	5	36	52	0.09
Total fat score	5	41	26	0.05
Total unsaturated fat	5	10	10	1.00
Total walking activity (MET.minutes/week)	5	1307	4158	0.07
Overall moderate-to-vigorous physical activity (MET.minutes/week)	5	3755	8303	0.10
Pedometer counts (steps per day)	5	5831	8555	<0.01

^a^Questionnaire items used a 10-point Likert scale.

## Discussion

### Main findings of this study

The results gave positive feedback for the feasibility of recruiting participants to a prevention programme in the general population, as demonstrated by our positive recruitment rate. Overall the two phases of the pilot study provided encouraging evidence for the effectiveness of the Let's Prevent programme at targeting illness perception and promoting health behaviour change.

Following the implementation of the second cycle of qualitative feedback, it was felt that the process had led to the development of an educational programme that was fit-for-purpose to be delivered to people with a diagnosis of IGR from an English-speaking population. Although some minor modifications to the education sessions were suggested during the second cycle of revisions and piloting, it was felt that further re-piloting would not be required after making the necessary changes. The effectiveness of this education package now required formal evaluation, which would form the basis of the RCT.

The Let's Prevent programme is currently being formally evaluated in an RCT running from 2009 to 2014. The study aims to show that we can provide long-term effective T2DM prevention, as well as improvements in secondary outcomes.^29^ The use of the MRC framework criteria to develop and refine the programme were essential to ensure that it would be ‘fit for purpose’ and inform a larger RCT. The iterative process involved the use of qualitative and quantitative data collection.

### What is already known on this topic?

Several large multicentre research studies from a number of different countries have confirmed that intensive lifestyle modification can prevent or delay the onset of T2DM.^8,9^ However, these studies involved frequent contact and support from healthcare professionals which is not always feasible in routine healthcare. The safe and effective transferability of these modifications to a clinical setting needs further exploration.

### What this study adds

This pilot study describes the development of a structured education, using sound learning theory principles and based on the MRC Framework criteria, and its refinement for implementation in an ongoing RCT. The processes that we followed in order to develop this programme can be replicated by other clinical researchers. The evaluation of both qualitative and quantitative data enhanced the content of the programme and ensured it could be used in different ethnic groups.

### Limitations of this study

One limitation of our study is the small sample size which was not sufficiently powered to accurately detect differences. Nevertheless it was felt that undertaking such analysis would give an indication on the direction of results. The ongoing RCT has been sufficiently powered to ensure that differences can be detected.

### Conclusions

Qualitative and quantitative data collected during piloting of the Let's Prevent programme suggested that intervention in a multi-ethnic population had the desired effect (change in short-term behaviour and illness beliefs) and demonstrated that the recruitment strategy and data collection methods used were feasible for implementation in an RCT setting. We will evaluate cost-effectiveness and we hope to be able to confirm that the programmes are feasible, reliable and suitable to be transferred from research into practice. Confirmation of transferability will, however, require implementation evaluation following the RCT. The RCT is the evaluation stage of the MRC framework.

## Funding

The project was supported by The NIHR Leicester-Loughborough Diet, Lifestyle and Physical Activity Biomedical Research Unit which is a partnership between University Hospitals of Leicester NHS Trust, Loughborough University and the University of Leicester and the Leicester Clinical Trials Unit.
